# Hemoglobin levels for determining anemia: new World Health Organization guidelines and adaptation of the national standard

**DOI:** 10.17843/rpmesp.2024.412.13894

**Published:** 2024-05-08

**Authors:** Gustavo F. Gonzales, Víctor Javier Suarez Moreno

**Affiliations:** 1 National Academy of Medicine, Lima, Peru. National Academy of Medicine Lima Peru; 2 Instituto Nacional de Salud, Lima, Peru. Instituto Nacional de Salud Lima Peru

Anemia is a condition in which the number of red blood cells is insufficient to transport the oxygen required by the cells for normal functioning. The hemoglobin value (g/dL) or hematocrit (%) is used to diagnose anemia. Iron deficiency, inflammation, deficiencies of other vitamins and micronutrients, hemoglobinopathies and hereditary diseases can cause anemia.

Estimates show that by 2021 there were 1.9 billion cases of anemia in the world, representing 24.3% of the world’s population of eight billion inhabitants for all ages ^1^. The populations most affected by anemia are those from tropical areas, low and low-middle income countries. An example are the high rates in sub-Saharan Africa and Southeast Asia, with figures exceeding 60% of the population of children aged 6 to 59 months, while the global percentage is 40%. Comparatively, the figures for anemia in this population group as of 2019 are 21% in Latin America and 29% in Peru.

The first warning call for iron deficiency anemia was made by the WHO in 1959, when they established a range of normality for children over 6-59 months, 6-12 years, male and female adults, and pregnant women ^2^. The values established in 1959 were modified in 1968 ^3^ and remained unchanged until 2024.

Peru is a country in which one third of the population lives in areas above 2,000 meters above sea level (masl). In 1968, hemoglobin (Hb) was adjusted for altitude with a value of 1 g/dL. Since 1989, in compliance with the proposal by the Centers for Disease Control and Prevention (CDC) in Atlanta ^4^, an adjustment equation was constructed for altitude over 1,000 meters above sea level; this adjustment is universally recommended for high altitude populations by the World Health Organization (WHO) ^5^. With this adjustment it was evident that high altitude populations have higher prevalence rates of anemia.

Several researchers have questioned the need to adjust hemoglobin for altitude in at least three continents (America, Africa and Asia) ^6^^-^^8^. Other researchers suggest redoing the adjustment formula based on new data collected throughout the world ^9^. A recent study conducted in Peru proposes that the CDC/WHO formula underestimates anemia in children aged 6 to 8 months residing in cities between 1400 and 2600 masl, while it may overestimate anemia between 3000 and 4300 masl ^10^.

The WHO has published a new guideline on cut-off points for defining anemia this year. The new recommendations have been formulated by conducting a systematic review using the GRADE (Recommendations assessment, development and evaluation) methodology. The guideline recommends an adjustment of Hb for altitude with a new equation that modifies the cut-off points from 500 masl. This new formula increases the adjustment factor for altitudes from 500 to 3000 masl and reduces it for altitudes above 3500 masl ^11^.

The guidelines also modified the hemoglobin cut-off points to define anemia in children aged 6 to 23 months (Hb=10.5 g/dL), in pregnant women in the second trimester (Hb=10.5 g/dL) and in residents of high-altitude areas. The WHO has not denied the fact that there may be new modifications in the future as more convincing research with greater certainty is developed ^11^.

On April 8, 2024, the Peruvian Ministry of Health (MINSA) promptly published Technical Standard NTS No. 213-MINSA/DGIESP-2024 ^12^, which adapts the new hemoglobin cut-off points proposed by the WHO.

These modifications occur at an important time, just when the reduction of anemia has stagnated, which would not allow meeting the targets of the Sustainable Development Goals (SDGs) by 2030. The modification of the Hb cut-off points should be taken into account for the new national surveys and the recalculation of previous years’ measurements should be evaluated to determine the trend in the prevalence of anemia.

The prevalence of chronic malnutrition evidenced by stunting in children < 5 years in Peru decreased significantly from 37% in 1992 to 13.1% in 2016. This decrease is considered a success attributed to a multisectoral government intervention, which included substantial investment in the expansion of anti-poverty policies and the introduction of comprehensive health insurance (SIS) for the poorest sectors of the population ^13^. This contrasted with the stagnation in reducing the prevalence of anemia despite intense efforts by national, regional and local governments.

The National Academy of Medicine (ANM), in its role of participating in the discussion and orientation of health problems and contributing to the progress of health sciences, to the quality of medical training and research on the medical-social reality of Peru, invites all educational institutions and research centers to disseminate the new guidelines and to develop the necessary research to reevaluate the prevalence of anemia in the different regions of the country, as well as to generate new information that will allow the WHO in the future to recommend new modifications of the Hb cut-off points for defining anemia. As a way to start working with the new guidelines, the ANM has formed a Temporary Committee of Experts which, together with members of the Academy, has incorporated researchers from the National School of Public Health (MINSA) and three universities to generate an institutional opinion on the new guidelines recommended by WHO.

The National Institute of Health (INS) in its mission to promote, develop and disseminate research, technologies and innovation in health that benefit the Peruvian population has developed since 2017 a webpage for anemia prevention in the “Prevention of Nutrient Deficiency” site, which was developed to provide technical and educational information related to the prevention and treatment of anemia in children from 6 to 35 months, pregnant and puerperal women. This site was designed to be used by professional and technical staff of first level health care facilities and includes a calculator for hemoglobin adjustment by altitude, and one for the calculation of iron supplement doses to be used according to age, weight, Hb, and altitude adjustment. These calculators should be updated promptly for effective use.

The reduction of anemia and the correct assessment for the diagnosis of anemia involves the participation of all of society as a whole, as well as national and international health agencies. Improved access to safe water and sanitation significantly reduces anemia by preventing many infectious diseases. Action against anemia should be comprehensive and not only focused on iron deficiency. Scientific research in particular areas such as the highlands and jungle which have different conditions that may favor its presence will be important to understand the different types of anemia in the country and how to address them.
