# Supplementary material for: Hemoglobin levels for determining anemia: new World Health Organization guidelines and adaptation of the national standard
Source: Rev Peru Med Exp Salud Publica. 2024 May 8;41(2):102–4. doi: 10.17843/rpmesp.2024.412.13894 (PMC11300700; doi:10.17843/rpmesp.2024.412.13894)
Supplement: Translation

# Translation: Niveles de hemoglobina para la determinación de la anemia: nueva guía de la Organización Mundial de la Salud y adecuación de la norma nacional

**Authors:** Gustavo F. Gonzales, Víctor Javier Suarez Moreno

**Affiliations:** 1 Academia Nacional de Medicina, Lima, Perú.; 2 Instituto Nacional de Salud, Lima, Perú.

La anemia se define como la condición en la cual el número de glóbulos rojos es insuficiente para transportar el oxígeno requerido por las células para su normal funcionamiento. Para su diagnóstico se utiliza el valor de la hemoglobina (g/dL) o del hematocrito (%). Las causas de la anemia pueden ser por deficiencia de hierro, inflamación, deficiencias de otras vitaminas y micronutrientes, hemoglobinopatías y enfermedades hereditarias.

Hacia el 2021, se estima que en mundo existían 1,900 millones de casos de anemia que representa para todas las edades el 24,3% de la población mundial que es de ocho mil millones de habitantes ^1^. Las poblaciones más afectadas por la anemia son las residentes en zonas tropicales, países de ingresos medios-bajos y bajos. Esto se observa en alto porcentaje en las zonas del África Subsahariana y del Sudeste Asiático y cuyas cifras superan el 60% de la población de niños de 6 a 59 meses en tanto que el valor global es de 40%. Comparativamente, las cifras de anemia en este grupo poblacional al 2019 es de 21% en América Latina y de 29% en Perú.

La primera llamada de alerta de la anemia por deficiencia de hierro fue realizada por la OMS en 1959, cuando establecen un rango de normalidad para niños mayores de 6 a 59 meses, 6-12 años, adultos varones y mujeres, y para gestantes ^2^. Los valores formulados en 1959 fueron luego modificados en 1968 ^3^ los cuales se mantuvieron invariables hasta el 2024.

El Perú se caracteriza por ser un país donde un tercio de la población vive en zonas por encima de 2,000 metros sobre el nivel del mar (msnm). En 1968 se decidió ajustar la hemoglobina (Hb) por la altura con un valor de 1 g/dL. Desde 1989, con la propuesta del Centers for Disease Control and Prevention (CDC) de Atlanta ^4^ se genera una ecuación de ajuste por nivel de altura desde los 1000 msnm y este ajuste es recomendado universalmente para poblaciones de altura por la Organización Mundial de la Salud (OMS) ^5^. Con este ajuste se observa que las poblaciones residentes en altura tienen una mayor prevalencia de anemia.

Varios investigadores han cuestionado la necesidad de ajustar la hemoglobina por la altura, por lo menos en tres continentes (América, África y Asia) ^6^^-^^8^. En tanto otros investigadores sugieren rehacer la fórmula de ajuste en base a los nuevos datos colectados a través del mundo ^9^. Un estudio reciente realizado en Perú propone que la formula CDC/OMS subestima la anemia en niños de 6 a 8 meses que residen en ciudades entre los 1400 y 2600 msnm, mientras que podría sobreestimar la anemia entre los 3000 y 4300 msnm ^10^.

La OMS ha publicado el presente año una nueva guía sobre los puntos de corte para definir anemia. Las nuevas recomendaciones se han formulado realizando una revisión sistemática de la literatura científica y utilizando la metodología GRADE (Recommendations assessment, development and evaluation). La guía recomienda un ajuste de Hb por altura con una nueva ecuación que modifica los puntos de corte desde los 500 msnm. Esta nueva fórmula aumenta el factor de ajuste para alturas de 500 a 3000 msnm y lo reduce para altitudes por encima de 3500 msnm ^11^.

La guía también ha hecho una modificación en los puntos de corte de la hemoglobina para definir anemia en niños de 6 a 23 meses (Hb=10,5 g/dL), en gestantes en el segundo trimestre (Hb=10,5 g/dL) y en los residentes en zonas de altura. La OMS no ha cerrado el hecho de que puede haber en el futuro nuevas modificaciones conforme se vayan desarrollando investigaciones más convincentes y de mayor certidumbre ^11^.

El Ministerio de Salud (MINSA) del Perú de manera célere el 8 de abril de 2024 ha publicado la Norma Técnica NTS N° 213-MINSA/DGIESP-2024 ^12^ con la cual se adecuan los nuevos puntos de corte de la hemoglobina propuestos por la OMS.

Estas modificaciones ocurren en un momento importante, justo cuando la reducción de la anemia se ha estancado y no permitiría cumplir con las metas de los Objetivos del Desarrollo Sostenible (ODS) al 2030. La modificación de los puntos de corte de la Hb, deberá tomarse en cuenta para las nuevas encuestas nacionales y evaluar el recálculo de las mediciones de años anteriores para observar la tendencia de la prevalencia de anemia.

La prevalencia de desnutrición crónica evidenciada por el retardo en el crecimiento en los niños < 5 años en Perú disminuyó significativamente de 37% en 1992 a 13,1% en 2016. Esta disminución se considera un caso de éxito atribuido a una intervención gubernamental multisectorial, que incluyó una inversión sustancial en la expansión de las políticas de lucha contra la pobreza y la introducción de un seguro de salud integral (SIS) para los sectores más pobres de la población ^13^. Esto contrastaba con el estancamiento en la reducción de la prevalencia de anemia a pesar de los intensos esfuerzos de los gobiernos nacionales, regionales y locales.

La Academia Nacional de Medicina (ANM) en su rol de participar en la discusión y orientación de los problemas de salud y de contribuir al progreso de las ciencias de la salud, al de la calidad de la formación médica y la investigación de la realidad médico-social del Perú invoca a todas las instituciones educativas y a los centros de investigación a difundir las nuevas guías y a desarrollar las investigaciones necesarias para revalorar las prevalencias de anemia en las diferentes regiones del país, así como generar nueva información que permita a la OMS en el futuro recomendar nuevas modificaciones en los puntos de corte de la Hb para definir anemia. Como una manera de iniciar el trabajo con las nuevas guías, la ANM ha conformado un Comité Temporal de Expertos donde con miembros de la Academia ha incorporado a investigadores de la Escuela Nacional de Salud Pública (MINSA), y de tres universidades para generar una opinión institucional sobre las nuevas guías recomendadas por OMS.

El Instituto Nacional de Salud (INS) en su misión de promover, desarrollar y difundir la investigación, las tecnologías y la innovación en salud que beneficien a la población peruana ha desarrollado desde 2017 su página para prevención de anemia en el portal de «Prevención de Deficiencia de Nutrientes» desarrollada para brindar información técnica y educativa relacionada a la prevención y tratamiento de la anemia en niños de 6 a 35 meses, mujeres gestantes y puérperas. Este portal se diseñó para ser utilizado por el personal profesional y técnico de los establecimientos de salud del primer nivel de atención e incluye una calculadora para ajuste de hemoglobina por altitud de residencia, y una para el cálculo de las dosis de suplemento de hierro a utilizar según edad, peso, nivel de Hb observada, y el ajuste por altitud. Estas calculadoras deberán ser actualizadas prontamente para su uso efectivo.

La reducción de la anemia y la valoración correcta para el diagnóstico de anemia involucra la participación de toda la sociedad en general, y a los organismos nacionales e internacionales que velan por la salud. La mejora en el acceso al agua potable y al desagüe reduce significativamente la anemia al evitar muchas enfermedades infecciosas. La acción contra la anemia debe ser integral y no solo enfocada a deficiencia de hierro. La investigación científica en zonas particulares como en la sierra y selva donde hay condiciones diferentes que pueden favorecer su presencia será importante para entender los diferentes tipos de presentación de anemia en el país y la manera de abordarlos.
